# Perioperative Management of Patients Undergoing Total Pancreatectomy with/without Islet Cell Autotransplantation: A Single Center Experience

**DOI:** 10.3390/jcm12123993

**Published:** 2023-06-12

**Authors:** Trista D. Reid, Supradeep S. Madduri, Chris B. Agala, Chengyu Weng, Sasha McEwan, Chirag S. Desai

**Affiliations:** 1 Department of Surgery, Division of Trauma and Acute Care Surgery, The University of North Carolina at Chapel Hill, Chapel Hill, NC 27599, USA; trista_reid@med.unc.edu (T.D.R.); sasha.mcewan@unchealth.unc.edu (S.M.); 2 School of Medicine, The University of North Carolina at Chapel Hill, Chapel Hill, NC 27599, USA; supradeep_madduri@med.unc.edu (S.S.M.); chengyu_weng@med.unc.edu (C.W.); 3 Department of Surgery, The University of North Carolina at Chapel Hill, Chapel Hill, NC 27599, USA; chris_agala@med.unc.edu; 4 Department of Surgery, Division of Abdominal Transplantation, The University of North Carolina at Chapel Hill, Chapel Hill, NC 27599, USA

**Keywords:** islet autotransplantation, total pancreatectomy, chronic pancreatitis, postoperative management, intensive care

## Abstract

Total pancreatectomy (TP) and islet cell autotransplantation (IAT) are complex operations that require intensive postoperative monitoring with standardized protocols. There are few studies detailing immediate perioperative management. The purpose of this study was to describe the perioperative management of post-pancreatectomy patients in the first week following surgery to guide clinicians in addressing salient points from different organ systems. This is a retrospective cohort review of prospectively collected data from September 2017 to September 2022 at a single institution, including patients 16 years and older who underwent TP or TPIAT for chronic pancreatitis. Patients were maintained on a heparin drip (TPIAT), insulin drip, and ketamine infusion. Primary outcomes were complications in the first 5 days following surgery and ICU length of stay (LOS). Secondary outcomes included overall LOS and mortality. Of 31 patients, 26 underwent TPIAT, and 5 underwent TP. Median ICU LOS was five days (IQR 4–6). The most common immediate postoperative complications were reintubation [*n* = 5 (16%)] and bleeding [*n* = 2 (6%)]. Median insulin drip use was 70 h (IQR 20–124). There was no mortality. Patients were extubated quickly and progressed well on the protocol. Immediate postoperative complications were generally minor and without long-term effects.

## 1. Introduction

Surgical intervention for chronic pancreatitis can be extremely challenging. Operative selection varies from parenchymal preserving operations, such as head or tail resection and duct drainage procedures, to total pancreatectomy (TP) with or without islet cell autotransplantation (IAT) [1]. TP with or without IAT has been used for a select group of patients with extensive disease throughout the entire pancreas. These operations have been critically evaluated due to the significant metabolic implications involved in removing the entire pancreas. Because of these metabolic considerations, most studies focus on longer-term outcomes in this population [2,3,4]. While some may briefly describe short-term complications, there are very few studies that expound on the immediate perioperative period in detail. 

In the immediate perioperative period, TP and TPIAT patients require various infusions, including insulin, which necessitate multidisciplinary management and increasingly require patients to be recovered in surgical intensive care units (SICU) per institutional protocols. Many ICU physicians may not be familiar or comfortable with the management of these patients as this is a very specific sub-population of abdominal transplant patients that require specialized attention. Hence, the purpose of this manuscript is to describe the ICU management and immediate postoperative outcomes in TP/IAT patients at our institution, providing guidance for ICU residents and attendings in caring for this patient population. This study outlines the ICU protocol for the first few days in the ICU, demonstrating the safety and effectiveness of the protocol, and subsequently discusses the intricacies of managing TP/IAT patients.

## 2. Methods

This study was approved by the University of North Carolina—Chapel Hill Institutional Review Board IRBIS 19-2591. This is a retrospective cohort review of prospectively collected data from September 2017 to September 2022 at a single quaternary care institution on all patients greater than 16 years of age undergoing TP or TPIAT for chronic pancreatitis. All TPIAT patients were maintained on a post-pancreatectomy protocol (Table 1). For TPIAT patients, the islet transplantation process involved islet isolation, followed by placement of a 6-0 prolene stay suture on the splenic vein stump with the introduction of a 14 French angiocatheter. Pre- and post-infusion portal vein pressure measurements were taken with an assembly, the islets were transfused, and the suture was tied down after the sheath was removed. 

Baseline demographic information was collected, including age, sex, race/ethnicity, body mass index (BMI), etiology of pancreatitis, comorbid conditions (including any renal disease; cardiac history including arrhythmias, congestive heart failure, prior myocardial infarction, and coronary artery disease; gastrointestinal disorders including inflammatory bowel disease (IBD), primary biliary cholangitis, and gastric ulcer disease; pulmonary disease including chronic obstructive pulmonary disease (COPD), obstructive sleep apnea (OSA), and asthma; stroke; and history of prior abdominal surgery), preoperative hemoglobin A1C, and whether islet cell transplantation was performed. Postoperative care was assessed, including length of stay (LOS) in days in the surgical intensive care unit (SICU) and overall hospital LOS in days; length of time in hours until extubation; length of time in hours until nasogastric tube (NGT) removal; use of narcotics and ketamine drips; mean laboratory values on postoperative day (POD) one, two, three, and seven (including white blood cell count, hemoglobin, platelets, sodium, potassium, creatinine, glucose, aspartate transaminase (AST), alanine transaminase (ALT), bilirubin, alkaline phosphatase, lactate, international normalized ratio (INR), activated partial thromboplastin clotting time (aPTT), and c-reactive peptide); percent of patients with positive cultures (separated into blood, respiratory, and urine cultures); and daily insulin dose on day one, two, three, and seven; and discharge disposition. 

The primary outcomes were ICU complications in the first five days following surgery and ICU LOS. These complications included bleeding (need for blood transfusion for known GI, intraabdominal, or retroperitoneal bleed, or re-operation), narcotic withdrawal symptoms (determined by transplant surgeon and intensivist using factors such as rigors, sweating, tachycardia, diarrhea, nausea/vomiting, dilated pupils, and encephalopathy), hypoglycemia less than 60 mg/dL requiring administration of dextrose, any recorded use of pressors or inotropes postoperatively, cardiac complications (arrythmia requiring medications, myocardial infarction, ventricular dysfunction postoperatively), delayed extubation greater than 24 h postoperatively, reintubation, delay in removal of NGT of more than 48 h, replacement of NGT, need for continuous renal replacement therapy (CRRT), infectious complications (documented positive blood, respiratory, or urine culture, or clinical findings of wound infection, computed tomography (CT) findings of abscess), deep tissue injury (DTI), thrombosis (deep venous thrombosis (DVT), pulmonary embolism (PE), HITT (heparin induced thrombocytopenia)), or cerebrovascular accident (CVA.) Secondary outcomes were overall LOS and in-hospital, three-month, and six-month mortality. 

TP and TPIAT patients were compared to determine if any issues arose specific to the islet cell transplantations; however, comparisons were limited given the low number of patients. The five TP patients who did not undergo IAT had pre-existing severe diabetes. Currently, we only offer TPIAT to diabetic patients if their stimulated c-peptide is significant enough to predict a good islet yield. Some management differences did exist between TPIAT and TP patients. Only the TPIAT patients receive the intensive insulin infusion with a goal glucose of 90–120 mg/dL. TP patients received standard endotool insulin infusions. TPIAT patients received a heparin infusion for portal vein thrombosis prevention and received laboratory draws every six hours for the first 48 h, carefully watching for evidence of portal venous thrombosis; however, TP without IAT patients received heparin prophylaxis only and did not require the same intensive six-hour laboratory value checks. With regards to extubation, islet cell surgeries are lengthy, patients are narcotic dependent, often the surgery progresses late into the evening, and our institutional protocol necessitates keeping them in the ICU postoperatively; thus, we most often leave the TPIAT patients intubated immediately postoperatively and perform a controlled extubation in the morning if safe. TP without IAT patients are often extubated in the OR unless the anesthesiologist or surgeon makes the decision to keep the patient intubated for clinical reasons.

All data were entered prospectively into Recap (Nashville, TN, USA. version 11.0.3, 2021). Statistical analysis was conducted using SAS (SAS Institute Inc 2013. Version 9.4M8, 2013, Cary, NC, USA). Medians and interquartile ranges (IQRs) were calculated for all patients, TP, and TPIAT, then TP and TPIAT patients were compared using Kruskal–Wallis, Fisher’s exact, and chi-squared tests where appropriate. 

## 3. Results

Thirty-one patients underwent TP from September 2017 to September 2022, with twenty-six (83.3%) of these undergoing TPIAT. Thirteen (42%) of the patients were male, with a median age of forty (IQR 28–48) (Table 2). Nineteen (61%) patients had prior abdominal surgery, including two patients who had had prior Whipple operations. The pancreatitis etiology was alcoholic in nine (29%) patients, genetic in eight (26%), divisum in eight (26%), and idiopathic in six (19%). Twenty-one (68%) of the patients were weaned off narcotic drips within 24 h, and the majority (*n* = 26, 84%) were maintained on ketamine drips after 24 h. The median length of time on an insulin drip was 70 h (IQR 20–124). 

Nine (29%) patients had a record of a glucose level of less than 60 mg/dL, but there were no adverse events related to hypoglycemia that occurred. Postoperative bleeding was noted in two (6%) patients, although five (16%) patients had acute blood loss anemia requiring a blood transfusion with no evidence of active bleeding (Table 3). Two (6%) patients had postoperative neurologic issues, including withdrawal symptoms in one and delirium in the other. One (3%) patient had an acute kidney injury, but no patients required dialysis. Nine (29%) patients remained intubated beyond 24 h, and five (16%) required reintubation. Only one (3%) patient appeared to experience hypoxia related to their pro-inflammatory state; the other reintubations were secondary to flash pulmonary edema, possibly attributed to medications in one patient, fluid overload in another, and related to agitation and mental status in two patients. Twenty-six (84%) had NGTs still in place after 48 h, and two patients (6%) briefly required NGT replacement. Two (6%) patients required the use of low-dose pressors in the postoperative period for approximately three hours each, and one (3%) had atrial fibrillation requiring medication management. There were no infectious complications, DTIs, thromboses, or DKA. 

There were not many differences between TP and TPIAT patients. TPIAT patients were more likely to be white (*n* = 23, 88% vs. *n* = 2, 40%, *p* = 0.03), less likely to have been a former smoker (*n* = 4, 15% vs. *n* = 3, 60%, *p* = 0.04), and less likely to have been on insulin preoperatively (*n* = 2, 8% *p* = 0.02). TPIAT patients were more likely to be maintained on a ketamine infusion for longer than 24 h (*n* = 24, 92% vs. *n* = 2, 40%, *p* = 0.02), more likely to stay intubated for greater than 24 h [median of 17 h (IQR 15–35) vs. 0 h (IQR 0–14), *p* = 0.02], and had a longer ICU LOS [median of 6 days (IQR 5–7) vs. 2 days (2–40, *p* = 0.01]. The median glucose for TPIAT patients was 117 (108–139) at day 1, 117 (102–128) at day 2, 116 (102–127) at day 3, and 134 (116–164) at day 7 (Table 4). The median glucose for TP patients was 139 (126–146) at day 1, 181 (136–185) at day 2, 182 (88–186) at day 3, and 151 (138–193) at day 7.

The median SICU LOS was five days (IQR 4–6), while the median hospital LOS was 12 (IQR 9–15) (Table 2). In- hospital, three-month and six-month mortality was 0 (0%), and all patients were discharged to home. Nine (34.6%) of the TPIAT patients were insulin independent by discharge from the ICU and the same by discharge from the hospital. 

## 4. Discussion

In this retrospective cohort examining the immediate postoperative management of TP and TPIAT patients, we observed that patients were extubated quickly and progressed well on the postoperative protocol. Immediate postoperative complications were generally minor and without long-term effects. Hypoglycemia, reintubation, and acute blood loss anemia were the most common issues noted in the first five days following surgery. 

While there are many studies evaluating long-term outcomes in TPIAT, there are very few studies that go in-depth into immediate postoperative management [4,5]. This vulnerable time period is critical for patients, as hypotension, derangements in glucose, anemia, and hypoxia can cause loss of islet function [4,6]. Patients are also at particular risk for both bleeding and thrombosis, given heparin protocols and increases in portal venous pressure [7,8]. Thus, close monitoring and a multidisciplinary approach are necessary.

### 4.1. Glycemic Control

One of the goals of TPIAT is to prevent the development of type 3c brittle diabetes [9]. Not all patients will achieve insulin independence following surgery; however, most of the patients will demonstrate c-peptide positivity at three years, a marker of continued islet cell function after TPIAT [10]. In the ICU, glucose management of TPIAT patients is more intensive than the goal of less than 180 mg/dL, which many ICU clinicians are familiar with [11,12]. Postoperative goals are to prevent hyperglycemia, which can damage islet cells, and hypoglycemia, which can be potentially life-threatening [6,13]. Hypoglycemia is common, with varied reports of 50% to more than 80% of patients experiencing hypoglycemia following IAT [14,15]. Of note, patients may not have symptoms of hypoglycemia until their glucose levels are below 40 mg/dL, highlighting the importance of monitoring in the postoperative period [15].

For glucose management, ICU insulin regimens generally target a glucose level of 80 to 120 via an insulin drip with the involvement of the endocrinologists, with glucose checks every hour for the first 48 h. Per our protocol, the insulin drip is initiated at a rate of 0.025–0.05 units/kg/hour and continued until POD four, then transitioned to a subcutaneous insulin regimen with assistance from endocrinology. At this point, the physicians may decrease glucose checks to every two to six hours (or before meals and at bedtime if the patient does not require insulin). Infusion of dextrose-containing fluid is limited to patients who have glucose levels of less than 70 mg/dL postoperatively, as intraoperative dextrose infusions have been linked with perioperative hyperglycemia [16].

Twenty-nine percent of the patients in our cohort had a glucose check below 60 mg/dL at some point in time; however, none of the patients experienced adverse effects from the hypoglycemia. All patients received 10% dextrose instead of 50% dextrose without adverse effects; thus, it may actually be safe and feasible to administer 10% dextrose and avoid larger swings in glucose levels. As with most institutions in the United States, at our institution, it is mandatory for patients on insulin infusions to stay in an ICU setting, given the need for frequent glucose checks. This requirement likely contributes to increased ICU lengths of stay in our patients [1]. We did have one patient who remained in the ICU, specifically beyond seven days, because of the insulin infusion. Further investigations are required to assess the safety and efficacy of transitioning patients to a lower level of care sooner. In this series, we report insulin independence of 34.6% at one year, which mirrors other studies [10].

### 4.2. Management of Thrombosis and Bleeding

Patients are at risk for thrombosis and bleeding after TPIAT, given that higher volume islet infusions can lead to increased portal pressures [17]. Heparin infusions may improve graft function in this population via postulated anti-inflammatory effects [18]. TP without IAT patients do not have this same risk of portal vein thrombosis; thus, they can be maintained on heparin prophylaxis only and do not require every six-hour lab checks. For TPIAT patients at our institution, we check aPTT every two hours until less than 40, then start a heparin infusion for an aPTT goal of 40–50 with aPTT checks every four hours. The timing of heparin administration is dependent on the surgeon and is determined based on intraoperative portal pressures. Liver Doppler ultrasound is performed on POD one, two, and five to assess portal venous flow [17]. Via this regimen, the cohort had no incidences of thrombosis. However, two patients in the cohort did experience bleeding and required reoperation for hemorrhage control; thus, the risk of thrombosis must be balanced with the risk of bleeding. This bleeding risk is comparable to an approximate 6% bleeding risk in other cohorts [8]. 

### 4.3. Management of Cardiac Function

In this cohort, few patients required the use of vasopressors postoperatively. We attempt to minimize the use of vasopressors because of the concern that splanchnic vasoconstriction with the resultant reduced portal flow can lead to an increased risk of complications such as segmental portal thrombus and reduction in graft function. This strategy must be balanced with fluid administration. Norepinephrine has some potential negative effects in that it is arrhythmogenic; can reduce blood flow, oxygenation, and hepatocellular function; and has greater arterial and vasoconstrictor effects than phenylephrine and other sympathomimetics [19,20]. However, there are very little data to support the use of one pressor over another in this population. Management of arrhythmias and other cardiac events should follow standard guidelines for ICU care. The one patient who experienced atrial fibrillation received metoprolol for rate control.

### 4.4. Respiratory Function

Prolonged intubation is not necessary, and many patients can be extubated within 24 h postoperatively after a standard spontaneous breathing trial, rapid shallow breathing index (RSBI) of less than 105, appropriate hemodynamics and mental status, as well as oxygen saturation/blood gas confirming appropriate oxygenation, ventilation, and pH of greater than 7.3. Approximately 70% of patients at our institution were extubated within 24 h. Instant blood-mediated inflammatory reaction (IBMIR) occurs hours to days after transplantation and can lead to acute respiratory distress syndrome (ARDS) [21]. IBMIR is often mitigated by anti-TNF therapies, such as etanercept; however, these therapies can also induce acute lung injury. Supportive care via ARDS strategies (judicious fluid management, low tidal volume < 6 mL/kg ventilation in intubated patients) is the mainstay of treatment. Only one patient appeared to experience a cytokine-mediated pulmonary reaction in our series; however, the reaction did not result in severe ARDS.

### 4.5. Neurologic and Pain Management

Reintubation from poor mental status occurred twice in our cohort, which highlights the importance of ensuring the patient is following commands prior to extubation, as well as the involvement of chronic pain and psychiatry in this population, as patients are at risk for delirium postoperatively given prior opiate use. The chronic pain consult is initiated prior to extubation, and patients are maintained on multimodal pain regimens, including a low-dose ketamine infusion. The starting dose for ketamine is variable, ranging from 0.05 to 0.5 mg/kg/hour, and is initiated by anesthesia in the operating room. The multimodal regimen additionally includes acetaminophen, non-steroidal anti-inflammatory drugs (NSAIDs), and gabapentinoids [22]. Only one patient in our cohort may have suffered from withdrawal symptoms during the ICU stay, highlighting the importance of involving the pain team (either chronic or acute) early in the course as patients will initially have higher pain needs than their home regimens and will also need weaning of their narcotics. 

### 4.6. Management of Nutrition

Gastric dysmotility is common following TP/TPIAT [23]. Delayed gastric emptying symptoms are some of the most commonly reported after TPIAT and can lead to increased readmission rates [1,24]. Narcotics can worsen these symptoms. Thus, many pancreatectomy protocols, including our institution’s, incorporate prokinetic agents such as either metoclopramide or erythromycin immediately postoperatively. We additionally schedule ondansetron in the immediate postoperative period to reduce nausea and schedule an acid-suppressing agent such as pantoprazole. The dietician is immediately consulted in the patient’s care on arrival to the ICU. Pancreatectomy patients lose the exocrine function of the pancreas; thus, enzymatic replacement therapy is also indicated. We begin pancrelipase once the patient is eating, generally 72,000 units with meals and 24,000–48,000 units with snacks. Of note, given the peak in enzymatic activity at 30 min and the likelihood of tubes less than 14 French clogging, oral enzymatic replacement is not usually recommended with enteral feeding [22]. Fortunately, we did not have many patients who required delayed NGT removal or tube feeding.

### 4.7. Limitations

The limitations of this study include the small sample size and that the data is derived from a single institution, which limits generalizability and the ability to draw conclusions from the data. The future application of this protocol to a larger number of patients could give us more information on its efficacy and potential drawbacks.

## 5. Conclusions

In conclusion, TP and TPIAT are being increasingly used as treatment options for pancreatitis. The complexity of these patients requires multiple teams to be familiar with perioperative management. Our experience described above helps familiarize various specialty physician groups with basic pathways and treatment algorithms.

## Figures and Tables

**Table 1:** TPIAT management protocol.

POD 0	Remain intubated Stat labs then every 6 h (ABG, CBC, CMP, PT/INR) Heparin drip for aPTT goal 40–50 Insulin drip with glucose checks every 1 h for glucose goal 80–120 mg/dL If glucose < 80 start D5 0.45% NS at the maintenance rate × 1 h If glucose > 80, start 0.9% NS at the maintenance rate NGT Low constant wall suction JP drain × 2: 1 in splenectomy bed, 1 in pancreatectomy bed Vancomycin, piperacillin/tazobactam, and micafungin ×7 days, or until OR and lab cultures result Metoproclamide, pantoprazole, SCDs Anakinra and etanercept for islet engraftment
POD 1	US liver Doppler Chronic pain team consult prior to extubation Extubate if appropriate Incentive spirometry every 1–2 h while awake As needed, psychiatry consult following extubation Out of bed towards the evening, PT/OT consults
POD 2	US liver Doppler Labs decreased to twice daily NGT removal or to gravity Remove Foley catheter If glucose is consistently 80–120 without insulin, then every 2 h on check
POD 3	Labs decreased to daily Consider discontinuing the heparin drip with a transition to enoxaparin Enoxaparin dosing strategy (prophylaxis vs. treatment) is to be determined by the surgeon based on portal pressure Anti-Xa 4 h following THIRD dose of enoxaparin Assess gastrointestinal function—remove NGT if still in place and consider starting clear fluids (sugar-free)
POD 4	Advance diet to carbohydrate-controlled (<30 g with meals, <15 g with snacks) with a dietitian consult Start pancrelipase per dietitian recommendation: generally, 72,000 units with meals and 24,000–48,000 units with snacks Transition insulin drip to subcutaneous insulin regimen per endocrinology with sliding scale every 6 h Decrease glucose checks to every 2–6 h (or preferably before meals, if not performed previously) If patient not requiring insulin, may decrease glucose checks to before meals and at bedtime (ACHS) Pain management per pain team: wean off PCA if possible
POD 5	US liver Doppler Adjust glucose checks to ACHS Convert all medications to PO, including intravenous pain medications

Abbreviations: POD—postoperative day; ABG—arterial blood gas; CBC—complete blood count; CMP—comprehensive metabolic panel; PT/INR—prothrombin test/international normalized ratio; aPTT—activated partial thromboplastin clotting time; mg/dL—milligrams per deciliter; D5—5% dextrose; NS—normal saline; NGT—nasogastric tube; JP—Jackson–Pratt; OR—operating room; SCD—sequential compression device; US—ultrasound; PT/OT—physical therapy/occupational therapy; g—gram; ACHS—before meals and at bedtime; PCA—patient-controlled analgesia; PO—per os.

**Table 2:** Demographic information on patients undergoing total pancreatectomy (TP) and total pancreatectomy with islet autotransplantation (TPIAT).

	All Patients *n* = 31	TPIAT *n* = 26	TP *n* = 5	*p*-Value
Age (median +/− IQR)	40 (28–48)	39.5 (28–48)	46 (30–46)	1.00
Sex *n* (% male)	13 (42%)	10(38%)	3(60%)	0.63
Pancreatitis Etiology n (%)				0.94
Idiopathic/unknown	6 (19%)	5 (19%)	1 (20%)	
Alcoholic	9 (29%)	7 (27%)	2 (40%)	
Genetic	8 (26%)	7 (27%)	1 (20%)	
Divisum	8 (26%)	7 (27%)	1 (20%)	
Race/ethnicity n (%)				**0.03**
Black	2 (6%)	1 (4%)	1 (20%)	
White	25 (81%)	23 (88%)	2 (40%)	
Hispanic	3 (10%)	1 (4%)	2 (40%)	
Asian	1 (3%)	1 (4%)	0	
Body Mass Index (median +/− IQR)	24.61 (22.62–29.5)	24.20 (22.62–29.50)	24.86 (24.84–26.66)	0.83
Comorbidities n (%)				
Any Renal disease	1 (3%)	1 (4%)	0	1.00
Cardiac	12 (39%)	10 (38%)	2 (40%)	1.00
Gastrointestinal	7 (23%)	5 (19%)	2 (40%)	0.56
Pulmonary	0 (0%)	0 (0%)	0 (0%)	NA
Prior Abdominal Surgery	19 (61%)	17 (65%)	2 (40%)	0.30
Other ^1^	2 (6%)	1 (4%)	1 (20%)	0.30
Diabetes n (%)				
Hemoglobin A1c > 6.5	12 (39%)	8 (31%)	4 (80%)	0.06
Pre-operative Insulin Use	5 (16%)	2 (8%)	3 (60%)	**0.02**
A1c 5.7–6.4	13 (42%)	12 (46%)	1 (20%)	0.29
A1c < 5.7	6 (19%)	6 (23%)	0	0.37
Smoking Status n (%)				**0.04**
Current	11 (35%)	9 (35%)	2 (40%)	
Former	7 (23%)	4 (15%)	3 (60%)	
Never	13 (42%)	13 (50%)	0	
Use of narcotic drip >24 h *n* (%)	10 (32%)	7 (27%)	3 (60%)	0.30
Use of ketamine drip >24 h *n* (%)	26 (84%)	24 (92%)	2 (40%)	**0.02**
Length of time on insulin drip median hours (IQR)	70 (20–124)	92.5 (35–129)	22 (20–25)	0.06
Length of time intubated median hours (IQR)	17 (14–35)	17 (15–35)	0 (0–14)	**0.02**
Length of time with NGT median hours (IQR)	66 (59–93)	64 (59–88)	88 (64–96)	0.21
SICU Length of Stay in days median (IQR)	5 (4–6)	6 (5–7)	2 (2–4)	**0.01**
Hospital Length of Stay in days median (IQR)	12 (9–15)	12 (9–14)	10 (9–21)	0.74
Discharge Disposition n (%)				
Home	31 100%)	26 (100%)	5 (100%)	NA
Skilled Nursing Facility or Long Term Acute Care Hospital	0 (0%)	0 (0%)	0 (0%)	

^1^ Specifically, aneurysm and transient ischemic attack. Abbreviations: IQR—interquartile range; NGT—nasogastric tube; SICU—surgical intensive care unit.

**Table 3:** Outcomes of patients undergoing total pancreatectomy (TP) and total pancreatectomy with islet autotransplantation (TPIAT) in the intensive care unit.

	All Patients *n* = 31 *n* (%)	TPIAT *n* = 26 *n* (%)	TP *n* = 5 *n* (%)
Postoperative Bleeding *n* (%)	2 (6%)	2 (8%)	0 (0%)
Acute Blood Loss Anemia (Required Transfusion) *n* (%)	5 (16%)	4 (15%)	1 (20%)
Neurologic ^1^ *n* (%)	2 (6%)	2 (8%)	0 (0%)
Blood Glucose <60 n (%)			
Number of total events	13	8	5
Number of patients with an episode <60	9 (29%)	7 (27%)	2 (40%)
Diabetic Ketoacidosis *n* (%)	0 (0%)	0 (0%)	0 (0%)
Use of Pressors ^2^ *n* (%)	2 (6%)	2 (8%)	0 (0%)
Renal n (%)			
AKI	1 (3%)	1 (4%)	0 (0%)
CRRT	0 (0%)	0 (0%)	0 (0%)
Any Cardiac ^3^ n (%)			
Arrhythmia	1 (3%)	1 (4%)	0 (0%)
Delayed Extubation ETT >24 h *n* (%)	9 (29%)	8 (31%)	1 (20%)
NGT >48 h *n* (%)	26 (84%)	21 (81%)	5 (100%)
Reintubation *n* (%)	5 (16%)	4 (13%)	1 (20%)
NGT Replacement *n* (%)	2 (6%)	1 (4%)	1 (20%)
Infectious ^4^ *n* (%)	0 (0%)	0 (0%)	0 (0%)
Deep Tissue Injury	0 (0%)	0 (0%)	0 (0%)
Thrombosis ^5^ *n* (%)	0 (0%)	0 (0%)	0 (0%)
Other ^6^ *n* (%)	1 (3%)	1 (4%)	0 (0%)

^1^ Withdrawal or delirium. ^2^ Norepinephrine, for less than three hours in each patient. ^3^ No episodes of heart failure or myocardial infarction. ^4^ Including positive blood, urine, or respiratory cultures; wound infections; or intraabdominal abscess. ^5^ Including pulmonary embolism, deep venous thrombosis, portal venous thrombosis. ^6^ Anakinra allergic reaction. Abbreviations: AKI—acute kidney injury; CRRT—continuous renal replacement therapy; ETT—endotracheal tube; NGT—nasogastric tube.

**Table 4:** Median laboratory values of patients undergoing pancreatectomy by days 1, 2, 3, and 7.

	Day 1		Day 2		Day 3		Day 7	
	TPIAT (*n* = 26)	TP (*n* = 5)	TPIAT (*n* = 26)	TP (*n* = 5)	TPIAT (*n* = 26)	TP (*n* = 5)	TPIAT (*n* = 26)	TP (*n* = 5)
Glucose	117 (108–139)	139 (126–146)	117 (102–128)	181 (136–185)	116 (102–127)	182 (88–186)	134 (116–164)	151 (138–193)
Hemoglobin	10.3 (9.7–10.7)	10.2 (9.2–11.1)	9.4 (8.8–10.3)	9.5 (8.7–11.2)	9.1 (8.7–9.9)	9.7 (9.4–11.1)	9.7 (8.9–10.1)	9.3 (9–11)
Platelets	183 (149–221)	276 (157–278)	196 (155–221)	269 (131–281)	227 (182–249)	328 (162–335)	499 (456–533)	682 (318–696)
Potassium	3.8 (3.7–3.98)	3.95 (3.75–4.1)	3.85 (3.66–3.98)	4.1 (3.9–4.1)	3.7 (3.55–3.88)	4.1 (3.6–4.2)	3.7 (3.5–3.9)	3.6 (3.5–3.85)
Creatinine	0.76 (0.54–0.89)	0.65 (0.47–0.71)	0.7 (0.54–0.84)	0.59 (0.55–0.61)	0.66 (0.53–0.8)	0.55 (0.46–0.69)	0.66 (0.51–0.87)	0.54 (0.49–0.67)
AST	99 (80–123)	219 (118–266)	85 (69–106)	184 (175–294)	56 (48–74)	119 (100 = 221)	28 (23–40)	40 (39–51)
ALT	70 (53–96)	130 (41–143)	64 (46–81)	142 (55–149)	53 (43–76)	119 (52–178)	40 (30–52)	67 (42–86)
Total Bilirubin	0.73 (0.47–1.3)	0.9 (0.7–1.5)	0.76 (0.45–1.2)	0.9 (0.6–0.95)	0.78 (0.5–1.0)	1.2 (1.1–1.2)	0.6 (0.4–0.9)	0.7 (0.5–0.8)
Alkaline Phosphatase	45 (31–54)	52 (51–58)	50 (40–55)	59 (51–83)	49 (43–57)	74 (56–90)	59 (48–67)	106 (59–129)
INR	1.3 (1.2–1.54)	1.26 (1.25–1.33)	1.25 (1.11–1.39)	1.27 (1.23–1.3)	1.19 (1.07–1.27)	1.15 (1.1–1.22)	1.15 (1.08–1.31)	1.18 (1.16–1.27)
C-reactive peptide	0.8 (0.3–1.2)	N/A	0.43 (0.2–0.9)	N/A	N/A	N/A	0.8 (0.2–1.4)	N/A

Abbreviations: TPIAT—total pancreatectomy islet cell autotransplantation; TP—total pancreatectomy; AST—aspartate transaminase; ALT—alanine transaminase; INR—international normalized ratio.

## Data Availability

Data are available on request but are not publicly archived due to privacy considerations.
